# Current status of laparoscopy teaching in gynecology and obstetrics medical residency in Brazil

**DOI:** 10.31744/einstein_journal/2024AO0458

**Published:** 2024-08-20

**Authors:** Carla Ferreira Kikuchi Fernandes, José Maria Cordeiro Ruano, Marair Gracio Ferreira Sartori, Renato Monteiro Zucchi, Marcelo Fiore Moutinho Capo

**Affiliations:** 1 Escola Paulista de Medicina Universidade Federal de São Paulo São Paulo SP Brazil Escola Paulista de Medicina, Universidade Federal de São Paulo, São Paulo, SP, Brazil.

**Keywords:** Education, medical, Internship and residency, Minimally invasive surgical procedures, Laparoscopy, Health knowledge, attitudes, practice, Surveys and questionnaires, Brazil

## Abstract

**Objective:**

To characterize laparoscopy teaching in Medical Residency Programs in Gynecology and Obstetrics in Brazil, and to evaluate preceptors’ characteristics in laparoscopy programs and map laparoscopic training practice scenarios.

**Methods:**

This descriptive cross-sectional study evaluated questionnaire responses from coordinators of the Medical Residency Programs in Gynecology and Obstetrics from 2019 to 2021.

**Results:**

The questionnaire was sent to 175 programs, and 90 responses were received (51.4%). From the 85 valid responses, it was noted that 67 programs had laparoscopic training. Of the 64 responses received regarding location, 32 replies (50%) indicated the Southeast of Brazil, particularly some country’s capitals. In 37.3% (n=25) of the cases, the program coordinator performed laparoscopy. The chief of the laparoscopy sector has advanced experience in most 52.5% (n=10) medical residency services; the preceptors also had advanced experience in 89.4% (n=59) of the services. Residents received laboratory training in 39.4% (n=26) of the services. In most cases, training was performed using a physical simulator. Of the 26 medical residency services with laparoscopy training outside the operating room, 80.8% (n=21) performed them as part of the curriculum, 61.5% (n=16) had a schedule for the same, and only 3.9% (n=1) were objectively evaluated.

**Conclusion:**

Laparoscopy teaching in Brazil is heterogeneous, with only a few programs offering any training in laparoscopy. The preceptors had advanced experience and participated in laboratory and operating room training. Only a few programs have their own laboratories or training centers, and most teaching programs do not plan to set up training centers.

## In Brief

This study characterized the state of laparoscopy teaching in Brazil through questionnaires sent to the coordinators of 175 Medical Residency Programs in Gynecology and Obstetrics. Laparoscopy teaching in Brazil is heterogeneous and inadequate. A few programs have their own training centers, and most teaching programs have no plans to set up one.

## Highlights

Only 20% of the medical residency programs have laparoscopic teaching.

Preceptors of the residency programs have advanced experience.

Most programs have no plan to set up a training center.

Resideny programs are concentrated in the Southeast region and in capitals.

## Graphical abstract

**Figure:**
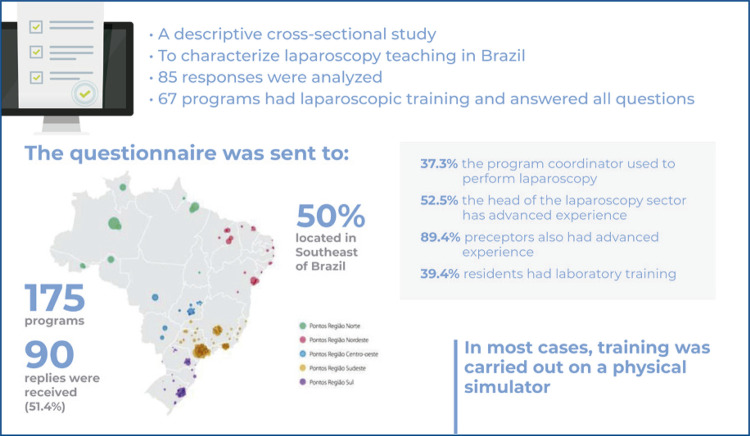

## INTRODUCTION

In 2020, Brazil hit the mark of 500,000 physicians, which is 2.28 physicians per 1,000 inhabitants. Almost 40% of Brazilian physicians work with no specialty.^( 1 )^Medical Residency remains the most important step after graduation and is the best method for training surgeons. Residents seek to develop both technical and non-technical skills such as leadership, ethics, and group work to guide their medical practice.^( 2 , 3 )^

In gynecology, minimally invasive surgical techniques such as laparoscopy, hysteroscopy, and robotic surgery are associated with better recovery, lower complication rates, shorter stays in hospitals, and an early return of patients to their activities.^( 4 , 5 )^ However, learning these technical skills requires guided teaching. In Brazil, laparoscopy learning starts in medical residency and follow the essential skills proposed by Brazilian Department of Education (MEC *- Ministério da Educação e Cultura* ) and endorsed by the Brazilian Federation of Obstetrics and Gynecology. Medical residency programs in obstetrics and gynecology must follow these guidelines to achieve a grid of competencies in laparoscopy (www.febrasgo.org.br/pt/matriz-de-competencias).^( 6 )^

In the United States, the Accreditation Council for Graduate Medical Education (ACGME) lays down the competencies that should be achieved in each specialty. The ACGME deems it essential to perform a hysterectomy in gynecological learning.^( 7 )^ There has been a reduction in the number of open and vaginal hysterectomies in the past few years, concurrently with an increase in laparoscopy hysterectomy. A decrease in the number of hysterectomies has been reported in countries such as Poland, Finland, and Brazil.^( 7 , 8 )^ Concomitant with the decrease in the number of surgeries, there has been a reduction in the medical residency course load.^( 4 , 8 , 9 )^

Teaching is challenging when there are decreased course loads and lower surgical demands. In recent decades, both physical and virtual simulators have been included in medical residency programs to reduce the learning curve, which has significantly improved teaching.^( 10 - 12 )^

Brazil is a large country with great cultural diversity, and socioeconomic inequalities exist within the federation. Therefore, it is critical to understand the status of teaching in medical residency programs to improve the quality of teaching for physicians and patient assistance.

Imparting both technical and non-technical skills for laparoscopy to junior surgeons requires specific training to enable them to attain competency before entering the operating room.^( 4 , 13 , 14 )^ To ensure effective training, institutions must have a structure and curricula in addition to well-prepared teachers.^( 4 )^ Thus, the present study sought to learn the current state of laparoscopy teaching in medical residencies in gynecology and obstetrics in Brazil to propose changes and guide services.

## OBJECTIVE

To characterize laparoscopy teaching in Medical Residency Programs in Gynecology and Obstetrics in Brazil, and to evaluate preceptors’ characteristics in laparoscopy programs and map laparoscopic training practice scenarios.

## METHODS

This descriptive cross-sectional study evaluated questionnaire responses received from the Coordinators of the MRPGOs, recognized by the MEC, from February 2019 to April 2021.

This study was approved by the Research Ethics Committee of the *Universidade Federal de São Paulo - UNIFESP* ; CAAE: 90426517.0.0000.5505; #4.130.554.

The study included questionnaires from respondents who had signed the free and informed consent terms. Duplicate questionnaires and those without signed free and informed consent terms were excluded.

Online questionnaires for this study were created using Google Forms. The questionnaire included 34 close-ended questions and eight open-ended questions about demography, professors, senior resident performance in the third year of training, teaching scenario, and curriculum in laparoscopy teaching, both in lab training and in the operating room during medical residency in gynecology.

All questions from the questionnaire were analyzed in a descriptive manner based on the number of answers, and percentages were calculated based on the total number of answers.

## RESULTS

Of the 212 MRPGO institutions in Brazil, 26 were inactive from 2019 to 2021, *i.e* ., there were open positions; however, the program did not have any residents during the study period. Thus, there were active 186 programs. Despite several attempts to contact the institutions, the questionnaire could not be sent to 11 institutions for various reasons, such as lack of answers to local phone calls or inactive emails.

The questionnaire was sent to 175 programs and 90 replies were received (51.4%). Five of the recorded responses were excluded. From the 85 replies analyzed, it was observed that 67 programs included laparoscopic training and answered all questions. The 18 programs without laparoscopic training answered only questions regarding their localization and future lab training, costs, and opinions on teaching.

### Demographic data

**Figure 1:**
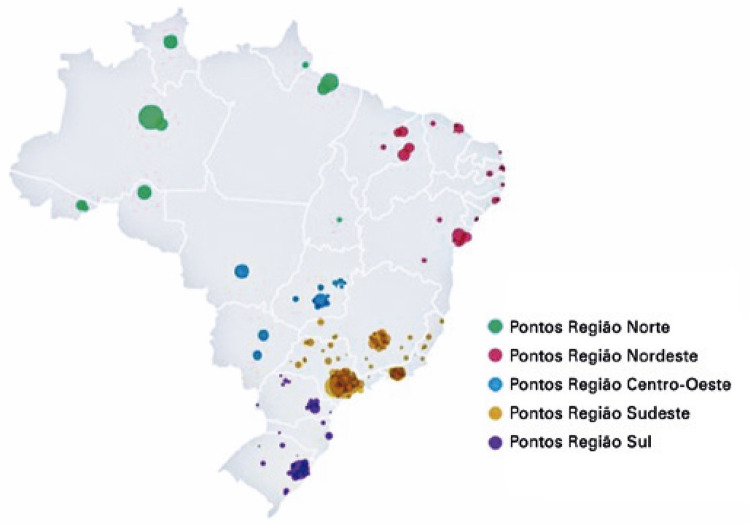
Geographic distribution of the 90 medical residency programs evaluated

Of the 67 programs, 63 answered about localization, and most were located in the southeastern region (50%, n=32), especially in the following capitals: São Paulo (15.9%, n=10), Belo Horizonte (8%, n=5), and Rio de Janeiro (6.4%, n=4). The regions with the second- and third-highest number of programs were the northeast (21.9%, n=14) and south (14.1%, n=9), respectively ( Figure 1 and Table 1 ).

**Table 1:** Location (country region) of MRPGO with training in laparoscopy

Region	Number of MRPGO	Percentage
Southeast	32	50.0
Northeast	14	21.9
South	9	14.1
Center West	6	9.4
North	2	3.2
Total answers	63	
No reply	4	

MRPGO: Medical Residency Programs in Gynecology and Obstetrics.

### Data regarding the coordinators of the MRPGOs

From the 67 replies, the median age of the coordinators was found to be 45.4 years, with a male predominance of 62.7% (n=42). Of the 67 coordinators, 37.3% (n=25) actively performed laparoscopy in a residency program service or private clinic and 46.3% (n=31) did not perform laparoscopy. Among the coordinators who performed laparoscopy (n=33), 30.3% (n=10) had been performing laparoscopy for over 15 years and 81.8% (n=27) performed more than 15 surgeries per year ( Table 2 ).

**Table 2:** Questions regarding the laparoscopy practice of coordinators of MRPGOs with laparoscopy training

	Southwest n (%)	Northeast n (%)	South n (%)	Center west n (%)	North n (%)	Not identified n (%)	Total n (%)
Q1. Do you perform laparoscopy in your institution or private clinic?							
Yes, I perform the laparoscopy myself	14 (43.8)	4 (28.6)	3 (33.3)	2 (33.3)	1 (33.3)	1 (33.3)	25 (37.3)
Yes, I do the laparoscopies with another qualified professional	6 (18.8)	2 (14.3)	0	1 (16.7)	1 (33.3)	1 (33.3)	11 (16.4)
No	12 (37.5)	8 (57.1)	6 (66.7)	3 (50)	1 (33.3)	1 (33.3)	31 (46.3)
Total	32 (100)	14 (100)	9 (100)	6 (100)	3 (100)	3 (100)	67 (100)
Q2. How many years you have been doing laparoscopy?							
Less than 5 years	2 (10.5)	1 (16.7)	0	0	1 (100)	2 (100)	6 (18.2)
Between 5 and 10 years	4 (21.1)	1 (16.7)	1 (33.3)	0	0	0	6 (18.2)
Between 10 and 15 years	8 (42.1)	1 (16.7)	1 (33.3)	1 (50)	0	0	11 (33.3)
More than 15 years	5 (26.3)	3 (50)	1 (33.3)	1 (50)	0	0	10 (30.3)
Total	19 (100)	6 (100)	3 (100)	2 (100)	1 (100)	2 (100)	33 (100)
Q3. How many laparoscopic surgeries do you perform per year?							
More than 5	1 (5.3)	1 (16.7)	0	0	0	0	2 (6.1)
Between 5 and 10	0	1 (16.7)	0	0	0	1 (50)	2 (6.1)
Between 10 and 15	0	1 (16.7)	1 (33.3)	0	0	0	2 (6.1)
More than 15	18 (94.7)	3 (50)	2 (66.7)	2 (100)	1 (100)	1 (50)	27 (81.8)
Total	19 (100)	6 (100)	3 (100)	2 (100)	1 (100)	2 (100)	33 (100)

### Data regarding the chief of the laparoscopy sector of MRPGOs

In 52.5% (n=10) of the 19 replies, the chief of the sector had been acting for more than 15 years. It was observed that 89.5% (n=18) performed more than 15 surgeries per year ( Table 3 ).

**Table 3:** Questions regarding the laparoscopy practice of the chiefs of the laparoscopy sectors of the MRPGOs

	Southwest n (%)	Northeast n (%)	South n (%)	Center west n (%)	North n (%)	Not identified n (%)	Total n (%)
Q4 How many years the person in charge has been performing laparoscopy?							
Less than 5 years	1 (14.3)	0	0	0	0	0	1 (5.3)
Between 5 and 10 years	0	1 (25)	1 (33.3)	0	0	1 (100)	3 (15.8)
Between 10 and 15 years	0	2 (50)	0	0	1 (100)	0	3 (15.8)
More than 15 years	4 (57.1)	1 (25)	2 (66.7)	3 (100)	0	0	10 (52.6)
Not known	2 (28.6)	0	0	0	0	0	2 (10.5)
Total	7 (100)	4 (100)	3 (100)	3 (100)	1 (100)	1 (100)	19 (100)
Q5 How many laparoscopic surgeries does the chief perform per year?							
Between 10 and 15	0	0	1 (33.3)	0	0	0	1 (5.3)
More than 15	6 (85.7)	4 (100)	2 (66.7)	3 (100)	1 (100)	1 (100)	17 (89.5)
Not known	1 (14.3)	0	0	0	0	0	1 (5.3)
Total	7 (100)	4 (100)	3 (100)	3 (100)	1 (100)	1 (100)	19 (100)

From the 66 replies received regarding laparoscopy service preceptors, it was found that there were advanced-level preceptors in 89.4% (n=59) of the services. In laparoscopic surgeries, there were advanced-level preceptors in 86.4% (n=57) and 71.2% (n=47) had more than 2 preceptors taking part in laparoscopic training.

### Data regarding training of residents in MRPGOs

#### In operating room

The coordinators of MRPGOs answered questions about the laparoscopic training of third-year residents in laparoscopic surgery during the medical residency program. Only one of the 67 services analyzed had a solely non-practical program; all others had practical training for laparoscopy.

In surgeries, 40.9% (n=27) of the residents acted as surgeons, 74.2% (n=49) acted as assistants, and 53% (n=53) acted as scrub nurses or camera operators. In this question, the residents could take different positions; thus, more than one option could be marked. It was observed that 43.9% (n=29) of the third-year residents participated in 11 or more surgeries per year, and 42.4% (n=28) participated in 6–10 surgeries per year.

#### In Lab training outside the operating room

Of the 66 practical training programs (one program with theoretical classes only), 39.4% (n=26) provided laparoscopy training outside the operating room with no patient contact during training ( Table 4 ). Of the 26 training programs in laboratories, 88.5% (n=23) had a black box or any other physical simulator. Regarding training length, seven of the 14 programs had 16–28 hours of training per year. Preceptors were present in 91.3% (n=21 of 23 services) of the training sessions.

**Table 4:** Does the service have training in laparoscopy outside the operation room (without patient participation)? Q12

	Southeast n (%)	Northeast n (%)	South n (%)	Center west n (%)	North n (%)	Not identified n (%)	Total n (%)
Yes	15 (48.4)	6 (42.7)	2 (22.2)	2 (33.3)	1 (33.3)	0	26 (39.4)
No	16 (51.6)	8 (57.1)	7 (77.8)	4 (66.75)	2 (66.7)	3 (100)	40 (60.6)
Total	31 (100)	14 (100)	9 (100)	6 (100)	3 (100)	3 (100)	66 (100)

Only seven (26.9%) of 26 programs had a virtual simulator. In 42.8% (n=3) of the cases, the training lasted more than 28 hours per year with preceptors.

Of the 26 programs, only one (3,8%) offered training on a human subject consisting of a 20-hour training per year with preceptors.

A total of 25 replies were received regarding the use of animals for training. Overall, 6 (24%) of the 25 programs had training on animals, and 50% (n=3) of these programs offered more than 16 hours per year with preceptors.

#### Training scenario

Of the 26 programs with training outside the operating room, 20 (76.9%) had partner labs or their on training centers.

Programs with and without training in laparoscopy were asked about setting up labs over the next 3 years. Of the 85 programs, 20 already had their own labs; therefore, this question was relevant to the remaining 65 programs without labs of which 52 answered the question. Of those, 28.9% (n=15) programs intended to set up a training center in the next 3 years, 28.9% (n=15) had no plan to do so, and 42.3% (n=22) might set up a lab.

The coordinators were asked about cost estimation, of which 52.9% (n=18) estimated an expenditure of $ 15,000.00 and 23.5% (n=8) had no cost estimation.

#### Training curriculum in laparoscopy in MRPGOs

All 26 programs with training in laparoscopy outside the operating room responded to questions regarding the curriculum; 80.8% (n=21) of them performed formal training as part of the curriculum, 61.5% (n=16) had a schedule for it in the medical residency course load, and 46.2% (n=12) had more than 20 hours of formal training per year.

Training was not evaluated in 38.5% (n=10) of the 26 programs, 26.9% (n=7) evaluated it subjectively, 30.8% (n=8) evaluated it both objectively and subjectively, and 3.9% (n=1) only evaluated training objectively.

All 67 programs were asked about the importance of laparoscopic training in laboratories before a resident performed surgery on a patient, and 97% (n=65) deemed it important. Of the 67 programs, 86.6% (n=58) selected “YES” concerning the need of an objective aptitude assessment before performing a surgery in a patient. Regarding the importance of laboratory training, 73.1% (n=49) deemed it important. All (n=67) coordinators agreed that training in a lab must be performed during medical residency at a specifically equipped center.

Finally, 97% (65) of the programs agreed that there must be a national consensus on laparoscopic surgery education in MRPGOs.

## DISCUSSION

The present study analyzed teaching laparoscopy in MRPGOs and observed that 20% of medical residency programs did not offer teaching in laparoscopy during medical residency. These data are alarming and suggest shortcomings in medical teaching.

The response rate (51.4%) for this study was considered satisfactory and matched survey response rates in health education literature.^( 1 , 2 , 4 , 14 - 17 )^ In order to achieve a satisfactory rate, the time for data collection was extended from 2019 to 2021. Some of the reasons for this extension were the COVID-19 pandemic, when many services reduced elective surgeries, and especially the difficulty in collecting data, as there are no centralized and computerized data.

Regarding the distribution of MRPGOs in Brazil, a concentration was observed in the southeastern region, in capital, and urban regions. In Brazil, most surgical specialties, including gynecology, are predominantly performed by males.^( 15 , 18 , 19 )^A limitation of this study was that we were unable to assess the location of the untrained group, as this was a non-mandatory response.

In medical residencies that provide laparoscopy training, the chief of the sector and residency preceptors have advanced experience in laparoscopy and often participate in training. Unfortunately, we received only limited responses for questions regarding the chief of the laparoscopic sector in the service, and we believe that few programs are organized. Residents actively participate in surgeries as surgeons, assistants, camera operators, or scrub nurses, despite the fact that the number of surgeries is insufficient to achieve any aptitude even for surgeries with low complexity.^( 1 , 6 , 11 , 20 )^

Several studies have shown that laboratory training outside the operating room in lab training must be formally included in the curriculum as part of the medical residency syllabus and offered frequently. The inclusion of lab training improves residents’ learning.^( 1 , 4 , 6 , 11 , 13 , 18 , 19 , 20 - 22 )^

From replies to plans for building a laboratory in the next 3 years, most programs did not plan to establish a laboratory in the next 3 years. Although most programs agree on the importance of a previous laboratory training medical residency and that aptitude training must be offered in a specific, properly equipped place, there is no plan to improve education in the short term. This might be related to a lack of investment and frequent change of coordinators. In Canada in 2015, almost 73% of the programs had formal teaching laboratories, 95% had access to technical skills laboratories, and 89% had non-technical skills training.^( 14 )^In the United States in 1997, studies predicted that by 2020, all cases would be assisted via laparoscopy, and the predictions came true.^( 23 )^In 2001, 29% of programs in the USA had laboratory training for laparoscopic skills, and by 2017, 86% had such training.^( 24 )^ In the present study, with data collected from 2019 to 2021, there was a delay of at least 4–10 years in relation to the training generally offered in North America. Considering the current lack of attention and investment in Brazil, the scenario is even worse.

Ideally, training assessment should be conducted objectively using a validated tool formally included in the curriculum. Tests and objective assessments such as the Global Operative Assessment of Laparoscopic Skills (GOALS), Objective Structured Assessment of Laparoscopic Salpingectomy (OSA-LS), and Fundamental Surgery of Laparoscopic Surgery (FSL) can motivate learning, encourage residents to study on their own, and take advantage of the educational opportunities available.^( 3 , 11 , 18 , 19 , 25 )^The present study investigated how residents’ assessments are performed; most of the coordinators agreed on the importance of an objective assessment, yet only 3.9% of the programs implement the same. To enter an operating room, residents must regard themselves as skilled in using their knowledge in a real environment.^( 4 , 20 , 21 , 26 - 30 )^

Some services, such as those in the United States, the Netherlands, Spain, and Finland, already have a specific curriculum aimed at a flexible program during medical residency according to personal interests or aptitudes. Early contact with a subspecialty is beneficial for professional schooling. A flexible curriculum does not imply that a subspecialty should be adopted at the end of residency to acquire knowledge and advanced skills.^( 13 , 30 )^

Surgical competence involves a combination of technical and nontechnical knowledge, decision-making, communication skills, and leadership. The greater the exposure to different teaching methods, the shorter the learning curve.^( 16 , 20 , 31 )^

This The present study objectively analyzed the characteristics of MRPGOs in laparoscopy teaching in Brazil. Several issues were identified, and it is possible to list some recommendations that could improve laparoscopy teaching in Brazil and other developing countries. The recommendations are as follows: 1) availability of lab training; 2) curriculum formally included as part of the medical residency course load with an objective validated assessment; 3) didactic teaching (in-person, e-learning, or video lessons); 4) presence of skilled preceptors to provide immediate feedback about training assessment and surgeries in patients; 5) flexible and specific curriculum; 6) adoption of a consistent approach to ensure high-quality education in all federation units; 7) building regional training centers, with public–private investment in different regions of the country to standardize teaching; 8) public–private partnerships with institutions with expertise in laparoscopy surgery.

## CONCLUSION

The teaching of laparoscopy in Medical Residency in Gynecology and Obstetrics in Brazil is heterogeneous and inadequate, with many programs not teaching laparoscopy at all. The preceptors of programs that offer training in laparoscopy have advanced experience and participate in laboratory and operating room training. Few programs have their own laboratories or training centers. Approximately one-third of the programs have no plans to set up a training center in the medium term.
